# Post-Synthetic Derivatization of Graphitic Carbon Nitride with Methanesulfonyl Chloride: Synthesis, Characterization and Photocatalysis

**DOI:** 10.3390/nano10020193

**Published:** 2020-01-22

**Authors:** Petr Praus, Aneta Smýkalová, Kryštof Foniok, Petr Velíšek, Daniel Cvejn, Jaroslav Žádný, Jan Storch

**Affiliations:** 1 Department of Chemistry, VŠB-Technical University of Ostrava, 700 80 Ostrava, Czech Republic; aneta.smykalova@vsb.cz (A.S.); krystof.foniok@vsb.cz (K.F.); 2 Institute of Environmental Technology, VŠB-Technical University of Ostrava, 708 00 Ostrava, Czech Republic; 3 Department of Advanced Nanomaterials and Organic Synthesis, Institute of Chemical Process Fundamentals, v.v.i., Czech Academy of Sciences, Rozvojová 1/135, 165 02 Prague 6, Czech Republic; velisek@icpf.cas.cz (P.V.); storchj@icpf.cas.cz (J.S.); 4 ENET Centre, VŠB-Technical University of Ostrava, 708 00 Ostrava, Czech Republic; daniel.cvejn@vsb.cz

**Keywords:** graphitic carbon nitride, derivatization, sulfur, mesyl chloride, photocatalysis

## Abstract

Bulk graphitic carbon nitride (CN) was synthetized by heating of melamine at 550 °C, and the exfoliated CN (ExCN) was prepared by heating of CN at 500 °C. Sulfur-doped CN was synthesized by heating of thiourea (S-CN) and by a novel procedure based on the post-synthetic derivatization of CN with methanesulfonyl (CH_3_SO_2_^−^) chloride (Mes-CN and Mes-ExCN). The obtained nanomaterials were investigated by common characterization methods and their photocatalytic activity was tested by means of the decomposition of acetic orange 7 (AO7) under ultraviolet A (UVA) irradiation. The content of sulfur in the modified CN decreased in the sequence of Mes-ExCN > Mes-CN > S-CN. The absorption of light decreased in the opposite manner, but no influence on the band gap energies was observed. The methanesulfonyl (mesyl) groups connected to primary and secondary amine groups were confirmed by high resolution mass spectrometry (HRMS). The photocatalytic activity decreased in the sequence of Mes-ExCN > ExCN > CN ≈ Mes-CN > S-CN. The highest activity of Mes-ExCN and ExCN was explained by the highest amounts of adsorbed Acetic Orange 7 (AO7). In addition, in the case of Mes-ExCN, chloride ions incorporated in the CN lattice enhanced the photocatalytic activity as well.

## 1. Introduction

Graphitic carbon nitride is a semiconducting nanomaterial that has been intensively studied during the last decade owing to its interesting properties such as high thermal, chemical, and photochemical stability [1,2]. Another important feature of carbon nitride (CN) is absorption of visible light as a result of band gap energy of 2.7 eV (459 nm), which is promising for various applications including solar cells’ fabrication [3], imaging, biotherapy, sensing of some compounds [4,5,6,7], and so on. However, most of the applications have been directed in the field of photocatalysis [8,9,10,11,12,13].

Besides these positive and useful properties, the serious shortcoming of CN is fast recombination of photoinduced electrons and holes, which has been solved by the formation of heterojunction composites with metal oxides, inorganic salts noble metals, and so on [13,14,15,16]. Recently, we have investigated the heterojunction composites of CN with TiO_2_ [17,18], WO_3_ [19,20], SnO_2_ [21], BiVO_4_ [22,23], BiOIO_3_ [24], ZnO [25], and graphene oxide [26]. Another possibility is the formation of heterojunction composites of pure and doped CN.

The doping with metal and/or non-metal elements allows us to tune band gap energy and to enhance absorption of visible light, physico-chemical, and photocatalytic properties. The topic of CN doping has been well described in several review papers [6,27,28]. Especially, doping with environmental-friendly non-metals, such as S, O, P, and N, is an interesting research topic that has been investigated at present [6,27,28]. There are a lot of papers dealing with the S-doping of CN in the literature. The common way is the synthesis of CN from sulfur-rich organic compounds, such as benzyl disulphide [29], thiourea [30,31,32,33,34,35,36], trithiocyanuric acid [37,38,39,40], elemental sulfur [41], H_2_S [42], and sulfuric acid [43].

Unlike these synthesis procedures from S-containing precursors, our new approach was based on the post-synthetic derivatization of already prepared bulk and exfoliated CN [44] with suitable highly reactive chemical agents having sulfur in their structure such as mesyl chloride. To the best of our knowledge, this is the first post-synthetic approach to S-dope CN, and no such synthetic strategy has been reported in the literature yet. Some modifications of CN with various organic compounds were referred to in the literature, but not in terms of the mesyl derivatization [45,46].

As already mentioned, thiourea is one of the often-used sulfur precursors and, therefore, such prepared S-doped CN was used as a comparative nanomaterial. All new nanomaterials were prepared and their properties were studied by means of common characterization and the photocatalytic decomposition of the commonly utilized dye Acid Orange (AO7).

## 2. Materials and Methods

### 2.1. Chemicals

All used chemicals were of analytical-reagent grade. Melamine and Acid Orange 7 were purchased from Sigma-Aldrich (Darmstadt, Germany), and thiourea was purchased from Merck (Darmstadt, Germany). Triethylamine (Penta, Czech Republic) and 1,4-dioxane (Lachner, Czech Republic) were dried by molecular sieves prior to reaction, and mesyl chlorid (Sigma-Aldrich) was used without additional purification. Distilled water was used for the preparation of all solutions and post-synthetic washing. The structures of melamine and AO7 are presented in Supplementary Materials (Figures S1 and S2).

### 2.2. Preparation of Bulk and S-Doped CN

Bulk CN was prepared by heating melamine at 550 °C for 4 h with the heating rate of 3 °C min^−1^ in a ceramic crucible with a lid (diameter 5 cm, 30 mL) in a muffle furnace. The crucible was cooled down out of the furnace to ambient temperature and then grounded in an agate mortar to a fine powder. S-doped bulk CN was prepared under same conditions as bulk CN. The only difference was that thiourea was used as a precursor instead of melamine. The content of sulfur in S-CN was determined by the X-ray fluorescence spectroscopy (XRF) (SPECTRO Xepos, SPECTRO Analytical Instruments GmbH, Kleve, Germany) and elemental analysis; see Table 1.

### 2.3. Exfoliation of Bulk CN

Exfoliated CN was performed by heating the bulk CN [44] in a thin layer on a ceramic plate (diameter 8 cm, 50 mL) at 500 °C in the muffle furnace for 2 h with the heating rate of 10 °C min^−1^. The ceramic plate with the product was cooled down to ambient temperature out of the oven.

### 2.4. Derivatization of Bulk and Exfoliated CN

The bulk and exfoliated CN (10 g) were suspended in dry 1,4-dioxane (100 mL) under inert atmosphere and triethylamine was added, and the suspension was cooled down in a cooling bath (water/ice) to ca. 10 °C. Then, mesyl chloride (50 mL, 0.73 mol) was added sequentially and the mixture was diluted by addition of 1,4-dioxane (50 mL). The cooling bath was removed and the reaction mixture was stirred at room temperature overnight. Then, the mixtures were quenched by addition of distilled water (ca. 20 mL) and the products were filtered off using a paper filter and washed with water (ca. 1000 mL) until neutral pH (according to pH-test strips). The content of sulfur in S-CN, Mes-CN, and Mes-ExCN was determined by the X-ray fluorescence spectroscopy (SPECTRO Xepos, SPECTRO Analytical Instruments GmbH Kleve, Germany) and elemental analysis (EA); see Table 1.

### 2.5. Ion-Exchange of Mes-ExCN with Hydroxide

First, 0.15 g of Mes-ExCN was added into 150 mL of NaOH (1 mmol/L) and stirred on a magnetic stirrer for 4 h. Then, the suspension was filtered through a membrane filter (0.6 μm), washed with 200 mL of water, and dried at 70 °C overnight. The solid product was used for the photocatalysis.

### 2.6. Elemental Analysis

The elemental analysis of C, N, and H in the prepared CN-based nanomaterials was performed using a Flash 2000 Elemental analyser (ThermoFisher Scientific, Waltham, MA, USA). The content of chlorine was determined by the Schöniger combustion method followed by the argentometric titration of chloride.

### 2.7. UV/Vis Diffuse Reflectance Spectroscopy

The UV/vis diffuse reflectance spectra (DRS) in the range of 220–1400 nm were recorded using a spectrophotometer Shimadzu UV-2600 (IRS-2600Plus) at laboratory temperature. Reflectance data were re-calculated to absorbance ones using Schuster–Kubelka–Munk’s equation *F*(*R*_∞_) as follows:

(1) $$F\left(R_{\infty }\right)=\frac{{\left(1-R_{\infty }\right)}^{2}}{2R_{\infty }},$$

where *R*_∞_ is the diffuse reflectance from a semi-infinite layer. The obtained DRS spectra were transformed to the dependencies of (*F*(*R*_∞_)·*hν*)^2^ on *hν* in order to obtain the optical band gap energies of the prepared nanomaterials.

### 2.8. FTIR-ATR Spectroscopy

Fourier transform infrared spectroscopy with (FTIR) was measured using the Nicolet iS50 device (Thermo Scientific, Waltham, MA, USA). The spectra were collected in the Attenuated total reflection (ATR) mode using a diamond ATR crystal. The spectra were collected in the wavenumber range of 400–4000 cm^−1^, and 32 scans were averaged. The ATR correction followed by baseline subtracting was applied on each spectrum using the OMNIC software (Waltham, MA USA).

### 2.9. X-Ray Diffraction

The phase composition and microstructural properties of the prepared nanomaterials were determined using the X-ray powder diffraction (XRD) technique. XRD patterns were obtained using a (Rigaku SmartLab diffractometer Rigaku, Tokyo, Japan) with a detector D/teX Ultra 250. A source of X-ray irradiation was a Co tube (CoKα, λ_1_ = 0.178892 nm, λ_2_ = 0.179278 nm) operated at 40 kV and 40 mA. The XRD patterns were collected in a 2θ range of 5–90° with a step size of 0.01° and speed of 0.5 deg min^−1^. The crystallite size *L* was calculated according to Scherrer´s equation for broadening *B*(2θ) (in radians) at a half maximum intensity (FWHM) of a diffraction peak as

(2) $$B\left(2\Theta \right)=\;\frac{K\lambda }{Lcos\Theta },$$

where *λ* is the wavelength of X-rays, θ is Bragg´s angle, and *K* is the constant equal to 0.94 for cube or 0.89 for spherical crystallites. In this study, *K* was rounded to 0.9.

### 2.10. TEM Analysis

Transmission electron microscopy (TEM) was performed with a JEOL 2100 microscope with (Jeol Ltd., Tokyo, Japan) a LaB6 electron gun. The accelerating voltage of 200 kV was applied. Micrographs were taken by a camera Tengra (EMSIS GmbH, Münster, Germany). For the TEM analysis, the samples were prepared by suspending in ethanol and were then sonicated for 5 min. One drop of this suspension was placed on a copper grid with a holey carbon film and was dried at room temperature.

### 2.11. Specific Surface Area Measurements

The specific surface area (SSA) of each nanomaterial was measured by a device SORPTOMATIC 1990 series (Thermo Scientific, Waltham, MA, USA). SSA was determined by the analysis of N_2_ adsorption isotherm at −196 °C by means of the Brunauer–Emmett–Teller (BET) method.

### 2.12. XPS Analysis

The superficial elemental analyses of the samples were carried out by means of an X-ray photoelectron spectrometer (XPS) ESCA 3400 (Kratos) with a base pressure in the analysis chamber of ~5.0 × 10^−7^ Pa. Electrons were excited with an Mg Kα radiation (*hν* = 1253.6 eV) generated at 12 kV and 10 mA. For all spectra, the Shirley background was subtracted. Peaks in all spectra ascribed to the sp^2^ hybridized nitrogen (C=N-C) were set to 398.8 eV as a charge correction.

### 2.13. High Resolution Mass Spectrometry (HRMS) Analysis

High resolution mass spectra were obtained by means of a MicrOtofIII spectrometer (Bruker Daltonik, Bremen, Germany) with Atmospheric pressure chemical ionization (APCI) ionization in a positive mode. Before the measurement, the samples were wetted with methanol and delivered into an APCI source in the solid state using a direct glass capillary inlet. The setting of the ion source was as follows: capillary—4000 V, end plate—500 V, corona—3000 nA, nebulizer (N_2_)—1 Bar, dry gas (N_2_)—4 L/min, temperature—drying 350 °C, and vaporizer—400 °C. The HRMS spectra were taken in the m/z range of 50 to 1550 Da with the low mass of 50 m/z and collision RF of 400 Vpp. The spectra were processed using the Compass Data Analysis 1.5 software (Bruker Daltonik, Bremen, Germany). The accurate mass scale was calibrated using ESI-L Low Conc. Tuning Mix (Agilent technologies, Santa Clara, CA, USA).

### 2.14. Photocatalytic Experiments

The photocatalytic activity was tested by means of the decomposition of AO7 in the concentration of 25 mg L^−1^ and this solution (150 mL) was placed into glass vessels: volume of 25 mL, height of 49 mm, and diameter of 32 mm. Under dark conditions, 10 mg of each nanomaterial was added. These suspensions were stirred for 60 min to reach adsorption equilibria and then irradiated under a UVA tube (368 nm, 0.94 mW cm^−2^) during times up to 120 min. The samples of 2 mL were taken and absorbances at 485 nm were measured by a UV/vis spectrometer Helios (Thermo Scientific, Waltham, MA, USA). The experiments verifying the influence of incorporated chloride ions were performed in glass vessels of 250 mL with a height of 52 mm and diameter of 90 mm.

## 3. Results

In order to incorporate a sulfur containing moiety into the structure of g-C_3_N_4_, highly reactive and cheap mesyl chloride was used instead of tosyl chloride, which is rather less reactive, or triflic chloride, which is more expensive. The physico-chemical properties of the CN-based nanomaterials were studied by means of several characterization methods. First, the nanomaterial elemental compositions were determined after their synthesis; see Table 1. Then, the light absorption was studied by UV/vis DRS; the structure was studied by XRD, FTIR-ATR, XPS, and HRMS. The texture properties were investigated by means of TEM and the physisorption of nitrogen. The photocatalytic properties were studied by means of the decomposition of AO7.

### 3.1. UV/Vis Diffuse Reflectance Spectrometry

**Figure 1:**
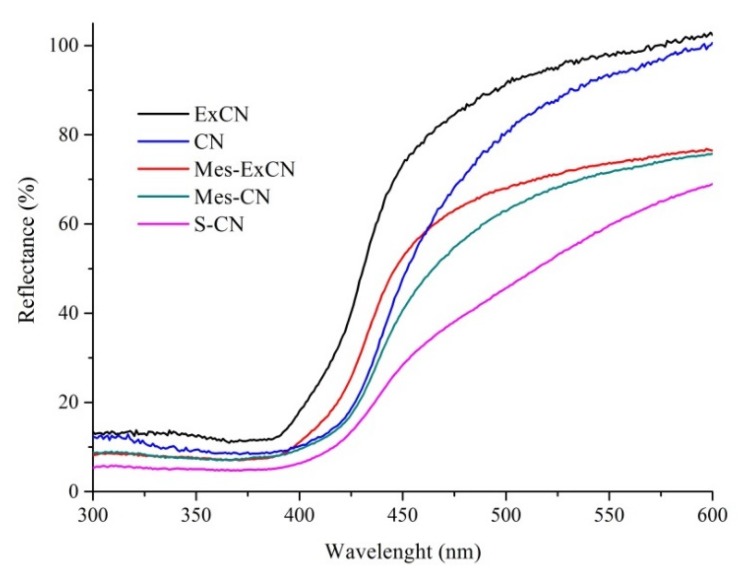
UV/vis diffuse reflectance spectra of carbon nitride (CN)-based nanomaterials.

The UV/vis DRS spectra shown in Figure 1 were recorded for us to observe light absorption properties and to determine the optical band gap energies of the prepared nanomaterials. For comparison, images of the prepared nanomaterials are demonstrated in Supplementary Materials (Figure S3) in order to display their real colors.

Figure 1 demonstrates that S-CN was the most-light absorbing sample and the absorption decreased in the sequence of S-CN > Mes-CN ˃ Mes-ExCN > CN > ExCN, which agrees with the nanomaterial colors displayed in Figure S3. The higher absorption of S-CN in comparison with CN was also referred to in the literature, for example, in the works of [32,35,36,39]. The derivatization of CN with mesyl chloride was supposed to incorporate new atom groups to heptazine units acting as chromophores. The resulting derivatized CN structures were discussed below based on other experimental results.

The optical band gap energies (hereinafter, the band gap energy) were evaluated by means of the commonly employed Tauc´s plot [47]

(3) $$\varepsilon h\nu =C{\left(h\nu -E_{g}\right)}^{p},$$

where *ε* is the molar extinction coefficient; *hν* is the energy of incident photons; *E_g_* is the band gap energy, *C* is a constant; and *p* is the power depending on the type of electron transition: *p* = 2 and *p* = ½ for direct and indirect semiconductors, respectively. In this study, *p* = ½ was used [48,49,50,51,52]. The evaluated band gap energies are summarized in Table 2. Both the direct synthesis as well as the derivatization led to insignificant changes of the band gap energies.

### 3.2. FTIR-ATR Spectrometry

**Figure 2:**
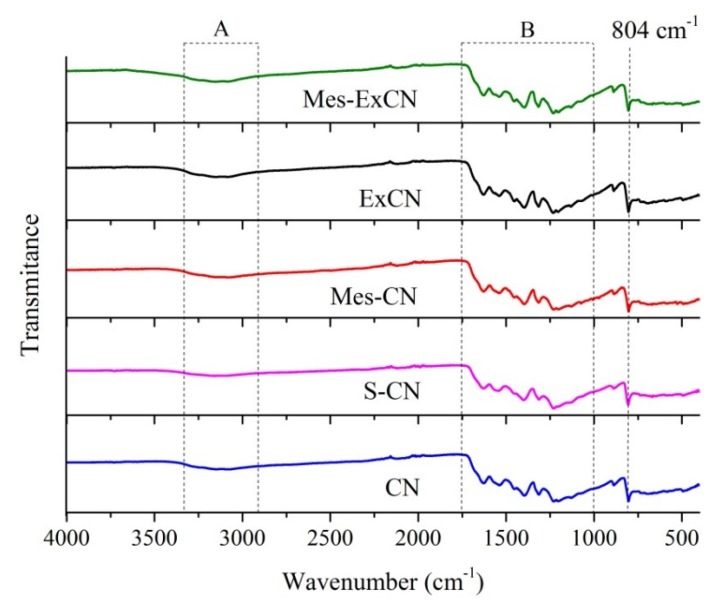
Fourier transform infrared spectroscopy (FTIR)-ATR spectra of CN-based nanomaterials.

The prepared nanomaterials were studied by FTIR-ATR spectrometry, as shown in Figure 2. The spectra contained broad spectral bands in the regions labelled as A and B. The spectral bands in region A can be attributed to the stretching vibrations of N–H bonds. The spectral bands in region B, such as 1232 cm^−1^, 1318 cm^−1^, 1399 cm^−1^, 1541 cm^−1^, and 1630 cm^−1^, are typically ascribed to the stretching vibrations of C=N and C–N bonds of heterocyclic rings. The medium band at 804 cm^−1^ can be attributed to the breathing mode of triazine units. All these spectra are typical for graphitic carbon nitride and their explanation can be found elsewhere, for example, in the works of [53,54,55,56].

Probably because of the low degree of derivatization, no significant differences between the FTIR-ATR spectra of CN and ExCN and Mes-CN and Mes-ExCN were observed. Thus, no evidence of the derivatization was brought.

### 3.3. XRD and TEM Analysis

**Figure 3:**
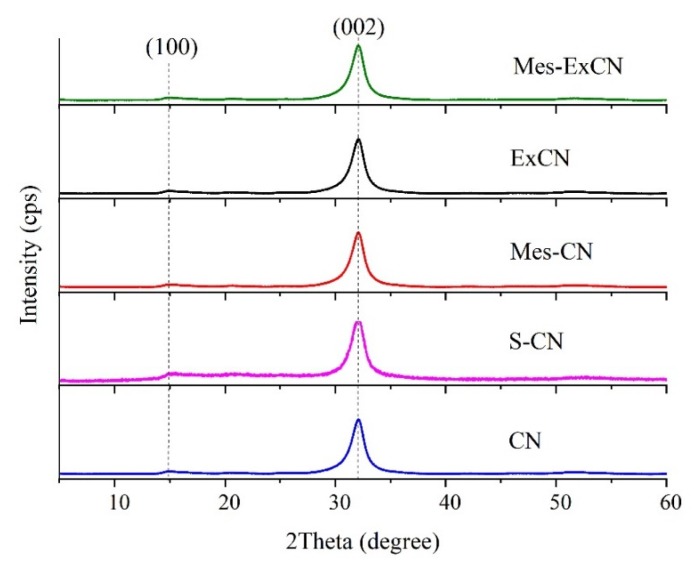
X-ray powder diffraction (XRD) patterns of CN-based nanomaterials.

The prepared nanomaterials were characterized by XRD and the patterns are shown in Figure 3. The two low intensive diffraction peaks at *2Θ* = 14.9° (*d_100_* = 0.690 nm) and 32.1° (*d_002_* = 0.324 nm) correspond to (100) and (002) diffractions, which can be attributed to the hexagonal phase of CN (JCPDS 87-1526). The stronger (002) diffraction peak is related to interlayer stacking of the (002) melem planes. The weaker (100) one is attributed to the in-plane ordering of nitrogen-linked heptazine units [57].

The crystallite sizes were calculated based on the (002) diffraction peaks by means of Scherrer´s Equation (2) and are summarized in Table 2. The *L_002_* values were nearly the same. This means that the sizes of diffracting domains as well as their interlayer distances were changed neither by the exfoliation nor the derivatization of CN. Unlike other authors [32,37,58], no change in the XRD pattern of S-CN as a result of the supposed substitution of nitrogen with sulfur in the CN lattice was observed.

**Figure 4:**
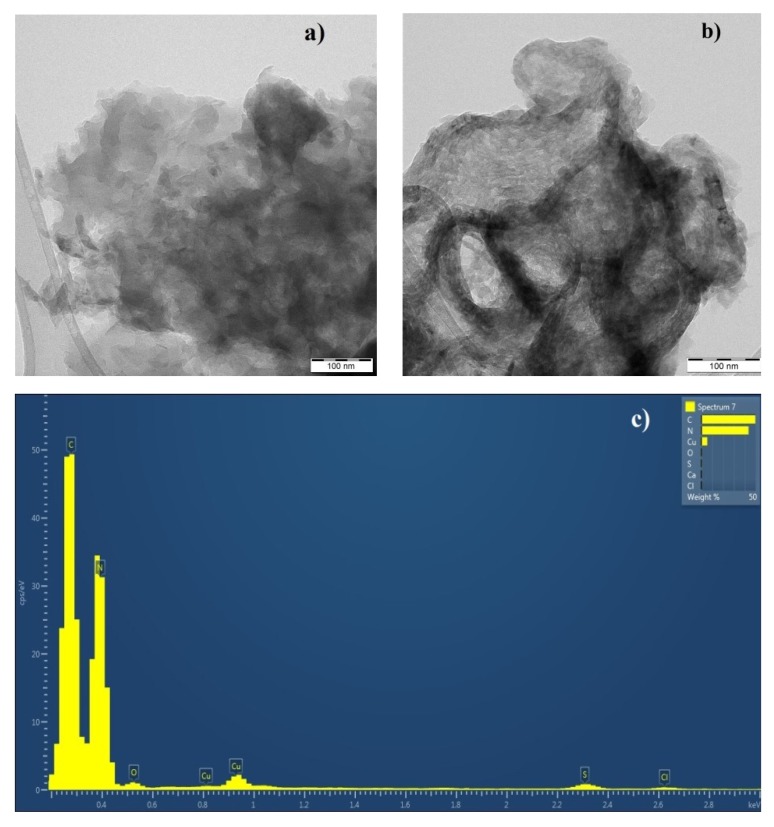
Transmission electron microscopy (TEM) micrographs of Mes-CN (**a**), Mes-ExCN (**b**), and EDX spectrum of Mes-ExCN (**c**).

The morphology of the nanomaterials was studied by means of transmission electron microscopy (TEM). The TEM micrographs are displayed in Figure 4. The complex CN structures were composed of flake-like sheets and snake-like shells. The flake-like structure was typical for bulk CN, regardless of the S-doping procedure. The shell-like structure as a result of the thermal exfoliation was observed for the ExCN (Figure 4a) as well as Mes-ExCN (Figure 4b). During the exfoliation, the flat nanosheets partially wrapped themselves into the shells and no derivatization effect was observed in the TEM micrographs. An energy dispersive X-ray (EDX) spectrum of Mes-ExCN is shown in Figure 4c. The presence of sulfur, chlorine, and oxygen is clearly demonstrated.

### 3.4. XPS Analysis

**Figure 5:**
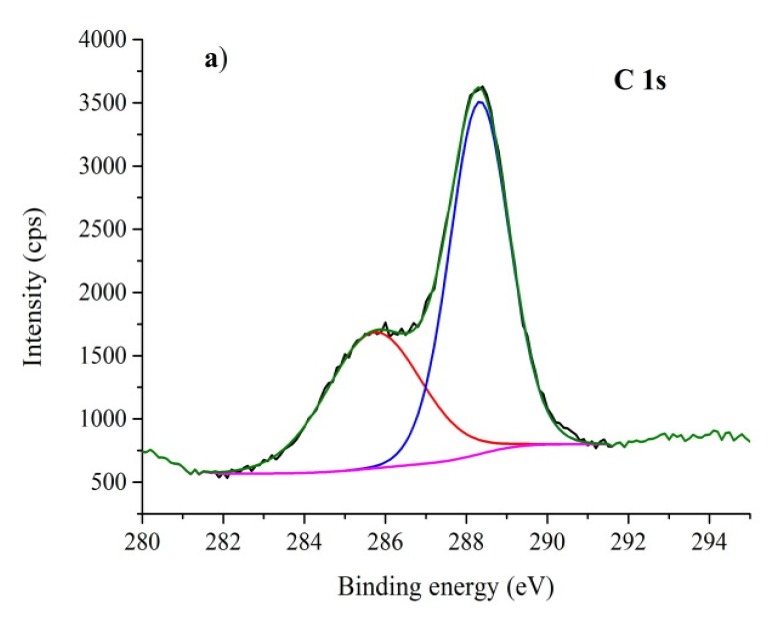

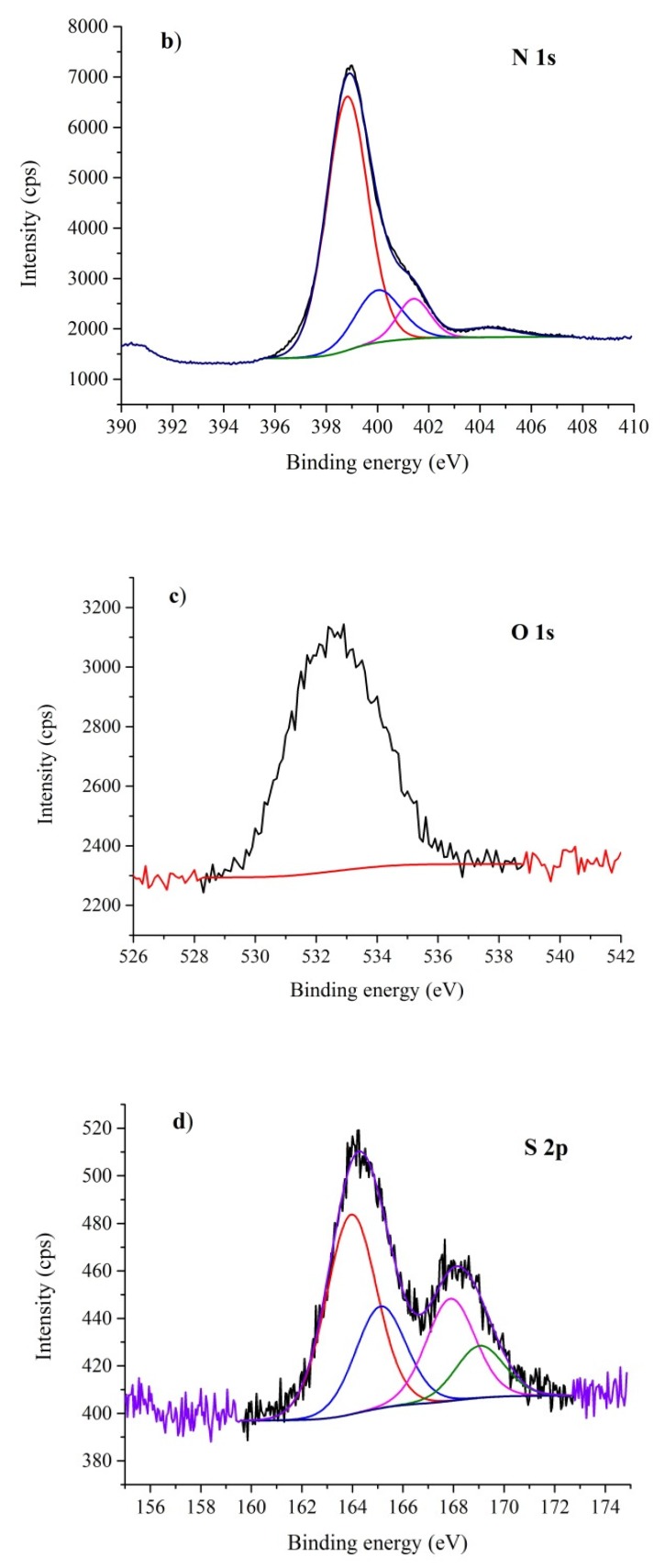
X-ray photoelectron spectrometer (XPS) spectra of Mes-ExCN. (**a**) C 1s, (**b**) N 1S, (**c**) O 1s, (**d**) S 2p.

The XPS analysis confirmed the presence of carbon, nitrogen, oxygen, and sulfur. The carbon 1s spectrum in Figure 5a shows two distinct peaks at 285.5 eV and 288.3 eV of binding energies. While the peak at 288.3 eV is clearly CN_3_ carbon typical for CN [59], the peak of 285.5 eV belongs to sp^2^ hybridized carbon of C=C or CN_2_ bonds [60,61]. Some portion of the 285.5 eV signal might be attributed to the carbon tape, which was used as a sample carrier. The ratio between the signals was roughly 3:1 in favour of 288.3 eV for CN and ExCN and 2:1 for S-CN. In the derivatised samples, the ratio decreased to 2:1 for Mes-ExCN and 1:1 for Mes-CN. This might suggest the exfoliation and derivatization effect onto g-C_3_N_4_. The signal of CH_3_, which occurred at about 284 eV, could not be equivocally detected by XPS owing to a low degree of derivatization in Mes-CN and Mes-ExCN and its overlap with the signal at 285.5 eV.

The shape of the nitrogen 1s peak (Figure 5b) was formed by the superposition of at least four signals at 398.8 eV, 400.0 eV, 401.4 eV, and 404.2 eV [62]. The dominant part of this spectrum was the pyridinic (triazinic) NC_2_ nitrogen signal, which corresponded to nitrogen atoms located at the edges of the melem subunits of g-C_3_N_4_. The signals of 400.0 eV and 401.4 eV were attributed to the NC_3_ nitrogen. The lower one was likely connected with bridging nitrogen atoms between melem structure cores (NC_3_^B^); the higher one was attributed to NC_3_^C^ nitrogen atoms in the centers of melem units. The theoretical ratios of NC_2_ and NC_3_ nitrogen 6:1 (in favor of N-C_2_ nitrogen for both NC_3_^B^ and NC_3_^C^) were found in no nanomaterials. That was probably because of the additional presence of NH^+^–C_2_ protonated nitrogen atoms (binding energy around 402 eV), bridging NH–C_2_ (binding energy around 399 eV), and marginal C–NH_2_ nitrogen (binding energy around 399 eV). Their capability to influence the shape of the spectra has to be taken into account [63].

The nitrogen region in the samples ExCN and Mes-ExCN showed the ratio between NC_2_ and NC_3_^B^ of roughly 4.5:1, suggesting the thermally exfoliated samples bearing more NH–C_2_ and marginal C–NH_2_ groups were not influenced by the derivatization. In Mes-CN, this ratio decreased to 3.3:1, while the pure CN had a ratio around 7.7:1, which suggests that, in the bulk nanomaterial, the derivatization led to increase of the amount of nitrogen atoms in NH–C_2_ or C–NH_2_. In comparison, the S-CN nanomaterial had ratios between both NC_2_ and NC_3_ identically around 10:1. Moreover, the overall portion in pyridine N–C_2_ nitrogen atoms decreased significantly in the CN nanomaterial after the derivatization (77 at.% vs. 66 at.% of all nitrogen atoms). This phenomenon was not observed in ExCN (71 at.% of all nitrogen atoms in both ExCN and Mes-ExCN). The most prolific nanomaterial in the overall NC_2_ portion is S-CN (81 at.% of all nitrogen atoms). All of the spectra have distinct broad peaks representing the binding energy of 404.2 eV and can be ascribed to π–π* (HOMO–LUMO) transition, that is, a shake-up line, a satellite peak [64].

The oxygen 1s broad peak (Figure 5c) around the binding energy 533 eV only confirmed its presence in the graphitic carbon structure and did not prove any further structure motive [65]. The sulfur 2p section of the spectrum in Figure 5d provided unsurprisingly no signal in the CN and ExCN samples. Interestingly, no significant sulfur 2p signal was recorded for the S-CN nanomaterial as well. On the other hand, in the cases of Mes-CN and Mes-ExCN, two types of sulfur incorporated in their structures were indicated. The first signal included a couple of 2p binding energies around 164 eV, suggesting sulfur in a low oxidation state (sulfide or thiol motive); the second one included another couple of 2p binding energies around 168 eV, suggesting a more oxidized form of sulfur [66], which could be attributed to mesyl groups. The sulfide XPS signal was explained by the presence of possible defects on the edge, which could promote electron delocalization, enabling the reduction of –SO_2_– groups [67]. The ratio between reduced and oxidized sulfur was inverse in the case of S-CN (approximately 1:3 in favor of oxidized sulfur) and S-ExCN (approximately 2:1 in favor of reduced sulfur).

### 3.5. HRMS Analysis

**Figure 6:**
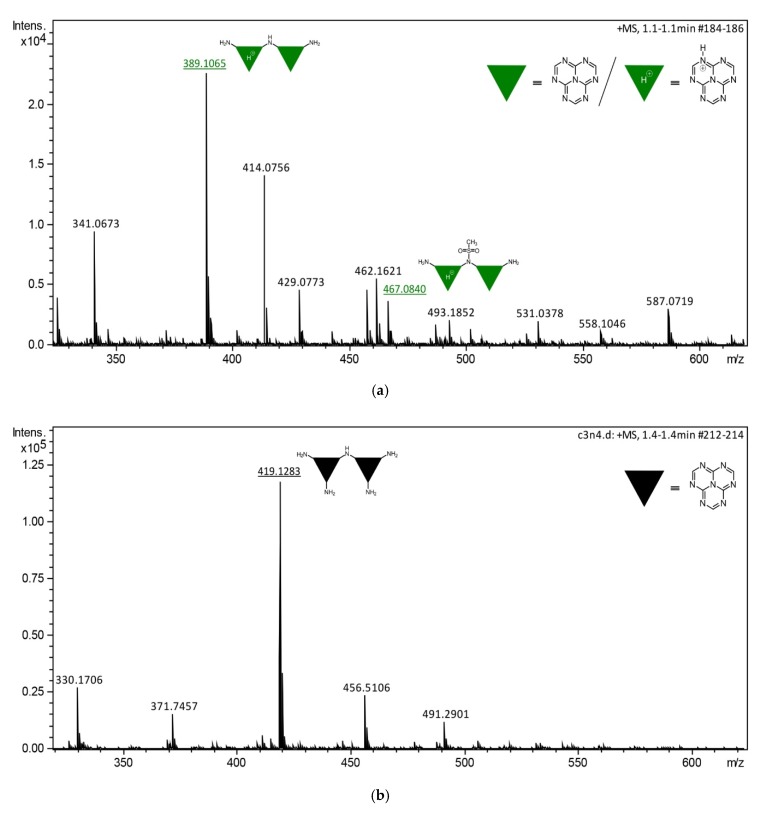
(**a**) Mass spectrometry (MS) analysis of Mes-ExCN. (**b**) MS analysis of ExCN.

The presence of mesyl functionalities upon the derivatization of ExCN was finally confirmed by measuring its mass spectra. It is obvious that, in the MS spectra of Mes-ExCN, several characteristic peaks were found proving the CH_3_SO_2_‒ functionality presence (Figure 6a), including the mass loss of 78.9854 (467.0840–389.1065 Da); corresponding structural moieties were depicted above the peaks. In comparison, Figure 6b shows the mass spectra prior to the derivatization of ExCN. Other structural moieties were not clarified within the accuracy of HRMS owing to the complexity of the investigated nanomaterials. The analysis evidenced the presence of CH_3_SO_2_^−^ moiety, and thus helped us to propose the structure of Mes-ExCN and its mostly presumable way of formation.

### 3.6. Overall Structure Theory

**Figure 7:**
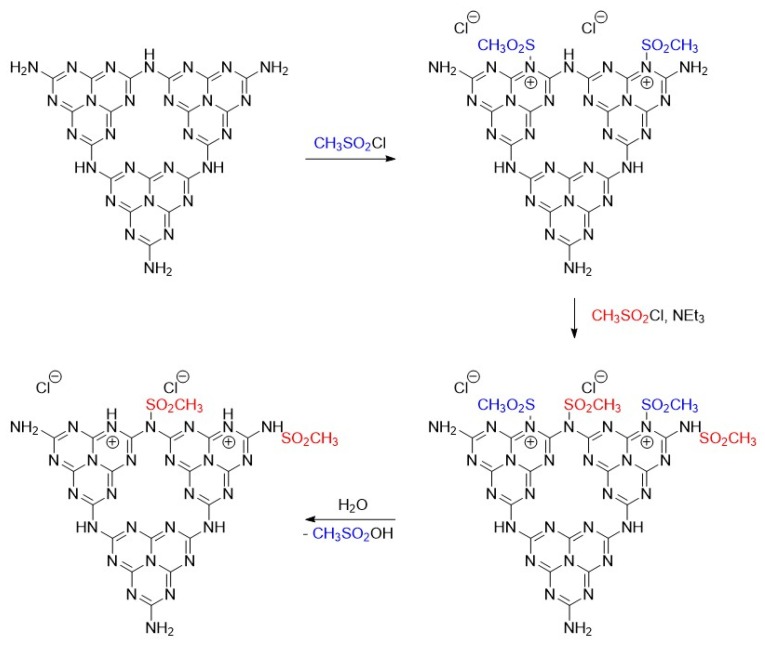
Proposed structure of CN after derivatization with mesyl chloride and its formation (only two melem units are depicted for simplicity of the scheme).

On the basis of information obtained from the XRD, FTIR-ATR, XPS, and HRMS results and considering the elemental composition of the nanomaterials, some characteristic structure motives of the post-synthetically derivatized nanomaterials were identified as demonstrated in Figure 7. The presence of chloride in the CN structure owing to bonding to NH^+^–C_2_ was confirmed by leaching of Mes-ExCN with the NaOH solution according to the procedure described in the Section 2.5. Chloride ions were exchanged with hydroxide ones and were determined by argentometric titration at 3.6 wt.%, which well agrees with the content of chlorine 3.33% found by EA; see Table 1. The incorporation of chloride into the CN lattice was described recently in the literature [68].

During the reaction of ExCN with the highly reactive mesyl chloride, the following reaction steps were suggested: (i) the reaction of mesyl chloride with the most nucleophilic triazine nitrogen to corresponding “pyridinium” species; (ii) the reaction of mesyl chloride with primary and secondary amine groups upon saturation of the most nucleophilic aromatic nitrogen atoms; and (iii) the hydrolysis of “pyridinium” moieties within the workup of reaction (Figure 7). This suggestion is based on the different nucleophilicity of triazine versus secondary (or primary) aromatic amines and also supports the presence of chloride ions in the resulting material.

### 3.7. Photocatalytic Activity

**Figure 8:**
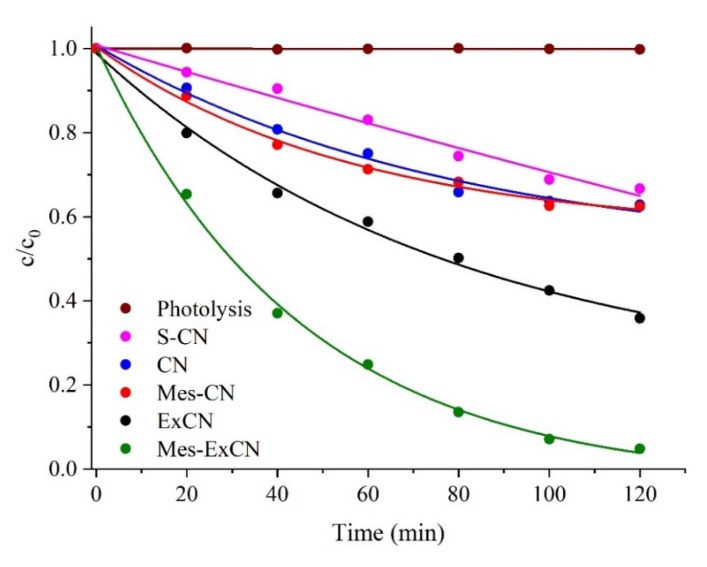
Photocatalytic decomposition of Acid Orange 7 (AO7) in aqueous dispersions of CN-based nanomaterials.

Heterogeneous photocatalytic reactions of a model compound, the azo-dye AO7 with radicals on the surface of the CN nanomaterials, can be described by the Langmuir–Hinshelwood model [69] for reaction rate *r* as follows

(4) $$r=-\frac{dc_{P}}{dt}=k\frac{K_{P}c_{P}}{1+K_{P}c_{P}+\sum K_{i}c_{i}}\frac{K_{R}\;c_{R}}{1+K_{R}c_{R}},$$

where *k* is the kinetic parameter; *K_P_*, *K_R_*, *K_i_*, *c_P_*, *c_R_*, and *c_i_* are the adsorption constants and the concentrations of the remaining AO7, radicals, and intermediates, respectively. If *c_R_* >> *c_P_* and Σ*K_i_c_i_* is neglected, then Equation (4) can be simplified to its most commonly used form:

(5) $$r=-\frac{dc_{P}}{dt}=k_{app}\frac{K_{P}c_{P}}{1+K_{P}c_{P}},$$

where *k_app_* is the apparent kinetic parameter depending on the irradiation intensity, mass, and nature of a solid phase (photocatalyst) and the concentration of the *R* radicals. Considering low values of adsorption constants and concentrations, we can assume that 1 + K_P_c_P_ ≈ 1, and then we obtain the common relationship:

(6) $$r=-\frac{dc_{P}}{dt}=k_{app}\frac{K_{P}c_{P}}{1+K_{P}c_{P}}=k_{obs}c_{P},$$

where *k_obs_* is the observed kinetic constant, supposing that the concentration of radicals is constant. The decomposition kinetic curves are shown in Figure 8. The observed kinetic constants were evaluated for all the tested nanomaterials and are summarized in Table 3. It can be concluded that the photocatalytic activity decreased in the sequence of Mes-ExCN ˃ ExCN ˃ CN ≈ Mes-CN ˃ S-CN.

In order to explain this photocatalytic behavior, the observed kinetics constant was compared with the specific surface area of the nanomaterials. The exfoliation increased the specific surface area of CN from 11 m^2^ g^−1^ to 90 m^2^ g^−1^ (Table 3). It is clear that the low *k_obs_* values of CN, Mes-CN, and S-CN can be related to the small SSA values, but this does not hold for the exfoliated nanomaterials.

The higher photocatalytic activity of S-CN than CN refeed in the previously mentioned papers was not confirmed. The reason is still a subject of further investigation.

**Figure 9:**
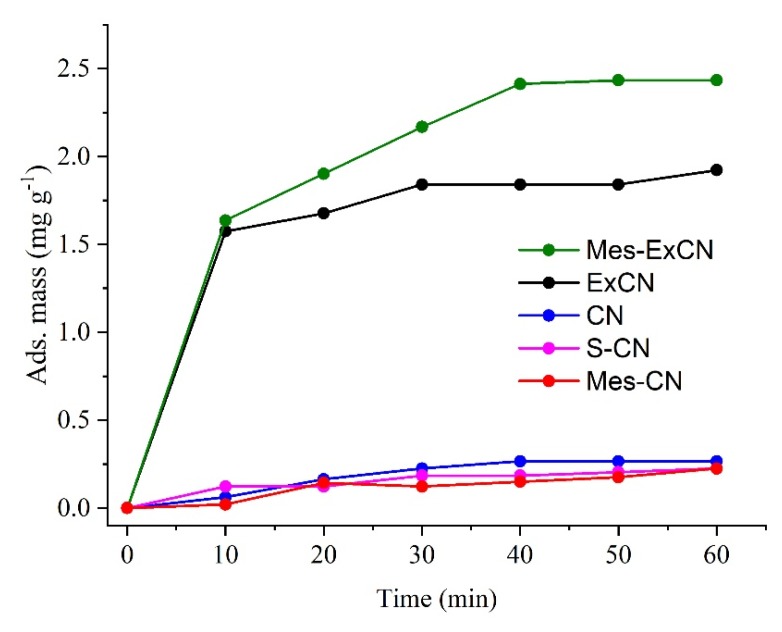
Adsorption of AO7 on CN-based nanomaterials depending on time.

The adsorption of AO7 on the nanomaterials was investigated under the same laboratory conditions, but in the dark. The adsorption plots depending on time are shown in Figure 9. Table 3 also summarizes the adsorbed mass of AO7 after 60 min. The adsorption equilibria for all adsorbents were reached during 40 min. The highest adsorbed mass of AO7 was observed for Mes-ExCN and ExCN; Mes-ExCN adsorbed more than ExCN. On the other hand, CN, S-CN, and Mes-CN adsorbed similarly very little. In case of the nanomaterials with the small SSA, the low mass of adsorbed AO7 was observed.

We can assume the adsorption was affected by SSA as well as by the derivatization, because new types of interactions between CN and AO7 appeared. Interactions of mesyl groups as well as van der Waals interactions of CN with AO7 molecules can be expected. On the basis of the EA data, the S/Cl ratio in Mes-CN and Mes-ExCN was only 0.2–0.25, which is consistent with the fact that protonation is much more effective than mesylation. Therefore, strong charge-assisted hydrogen-bonding interactions formed by means of NH^+^ groups of CN and SO_3_^−^ ones of AO7 can be expected as well.

**Figure 10:**
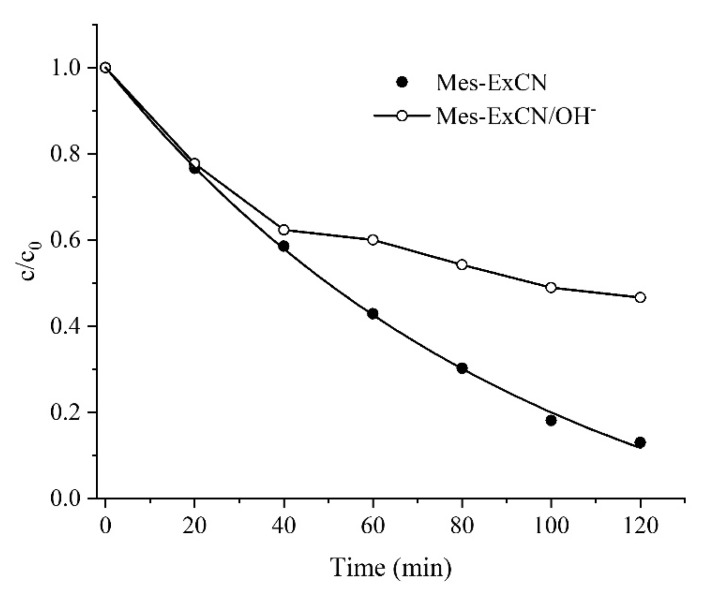
Photocatalytic decomposition of AO7 in aqueous dispersions of original MeS-ExCN and MeS-ExCN exchanged with hydroxide (Mes-ExCN/OH^−^).

The effect of chloride ions incorporated in the CN lattice on the photocatalytic activity was also investigated. As mentioned in Section 3.6, chloride ions in MeS-ExCN were exchanged with hydroxide ones (see Section 2.5), and such a prepared nanomaterial was tested for the photocatalytic decomposition of AO7 (see Section 2.14). The obtained kinetic curves are shown in Figure 10. It is obvious that the photocatalytic activity of MeS-ExCN with chloride and with hydroxide ions (MeS-ExCN/OH^−^) was similar up to 40 min. Then, the activity of MeS-ExCN/OH^−^ decreased significantly. The reason is unclear, but the exchange of hydroxide ions with anions resulting from the decomposition of AO7, such as sulfate and salts of organic acids [70,71], could be possible. Chloride ions were shown to play an important role in the photocatalytic decomposition of AO7, which is supported by results already reported in the literature [68,72]. Their activity was explained by (i) enhancement of electronic delocalization owing to extending the local 2D π-conjugated system of CN and (ii) up-shift of the conduction band of CN providing a stronger reduction ability of photogenerated electrons and oxidation ability of formed radicals.

Therefore, we can summarize that the enhanced photocatalytic activity of Mes-ExCN can be explained by the combination of higher adsorption ability and the activity of chloride ions incorporated in the derivatized nanomaterials.

## 4. Conclusions

Graphitic carbon nitride was synthetized by heating of melamine at 550 °C for 4 h. The exfoliated CN was then prepared by heating of the bulk one at 500 °C for 2 h. Together with these nanomaterials, S-CN was synthesized by heating of thiourea and bulk and exfoliated CN were derivatized with mesyl chloride. All these nanomaterials were investigated by several characterization methods and their photocatalytic activity was tested by means of the decomposition of AO7. S-CN served for comparison in this study.

The content of sulfur decreased in the sequence of Mes-ExCN ˃ Mes-CN > S-CN. The absorption of light decreased in the opposite manner. The influence of the sulfur content on the band gap energies was not found because the exfoliation effect indicated by the increase of specific surface area (from 11 m^2^ g^−1^ to 90 m^2^ g^−1^) played an important role as well. The nanomaterials were further examined by FTIR-ATR, XRD, TEM, XPS, and HRMS. Unlike S-CN, in which sulfur has been supposed to substitute nitrogen in the CN lattice, sulfur in Mes-ExCN and Mes-CN was confirmed to be a part of mesyl moieties connecting to primary and secondary amine. Chlorine retrained in the structure in the form of chloride ions, which were determined by argentometry.

The photocatalytic activity decreased in the sequence of Mes-ExCN ˃ ExCN ˃ CN ≈ Mes-CN ˃ S-CN. The highest activity of Mes-ExCN and ExCN was confirmed by the highest amounts of adsorbed AO7 to these nanomaterials and by the presence of chloride ions in the MeS-ExCN lattice.

The post-synthetic derivatization was likely the first approach to obtain doped graphitic carbon nitride. Future research will be focused on the synthesis and characterization of CN-based nanomaterials doped with other elements by means of some post-synthetic reactions. Their applications, especially in environmental photocatalysis, will be investigated.

## Figures and Tables

**Table 1:** Elemental composition of carbon nitride (CN)-based nanomaterials. XRF, X-ray fluorescence spectroscopy; EA, elemental analysis; CI, confidence interval.

Nanomaterials	C (%)	H (%)	N (%)	S (XRF/EA) (%)	Cl (%)
CN	34.54	1.72	62.31	---	---
ExCN	33.42	1.87	60.42	---	---
S-CN	34.06	1.74	61.15	0.22/n.d.	---
Mes-CN	35.43	2.20	56.36	0.26/0.34	1.43
Mes-ExCN	33.39	1.80	60.20	0.56/0.70	3.33

Note: n.d.—not determined.

**Table 2:** Evaluated values of band gap energy and crystallite size of prepared nanomaterials.

Nanomaterial	E_g_ (eV)	L_002_ (nm)
CN	2.69	6.6
ExCN	2.77	6.6
S-CN	2.63	6.6
Mes-CN	2.66	6.6
Mes-ExCN	2.73	6.7

**Table 3:** Observed kinetic constants and specific surface area (SSA) of CN-based nanomaterials. AO7, Acid Orange 7.

Nanomaterial	k_obs_ × 10^−3^ (min^−1^)	SSA (m^2^∙g^−1^)	Ads. AO7 (mg g^−1^)
CN	22.2 ± 3.0	11	0.27
ExCN	35.7 ± 3.3	90	1.9
S-CN	15.8 ± 2.4	20	0.22
Mes-CN	22.2 ± 5.3	8	0.22
Mes-ExCN	113 ± 9	67	2.4

## Supplementary Materials

The following are available online at https://www.mdpi.com/2079-4991/10/2/193/s1, Figure S1: Structure of melamine; Figure S2: Structure of Acid Orange 7; Figure S3: Images of prepared nanomaterials.
