# Supplementary material for: Post-Synthetic Derivatization of Graphitic Carbon Nitride with Methanesulfonyl Chloride: Synthesis, Characterization and Photocatalysis
Source: Nanomaterials (Basel). 2020 Jan 22;10(2):193. doi: 10.3390/nano10020193 (PMC7074974; doi:10.3390/nano10020193)
Supplement: Supplementary file 1 [file nanomaterials-10-00193-s001.pdf]

Supplementary materials

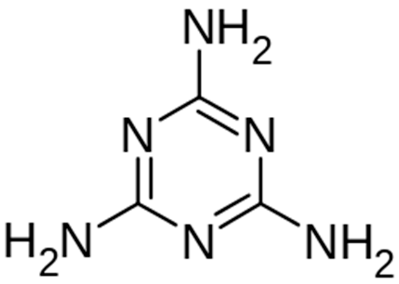

Figure S1. Structure of melamine

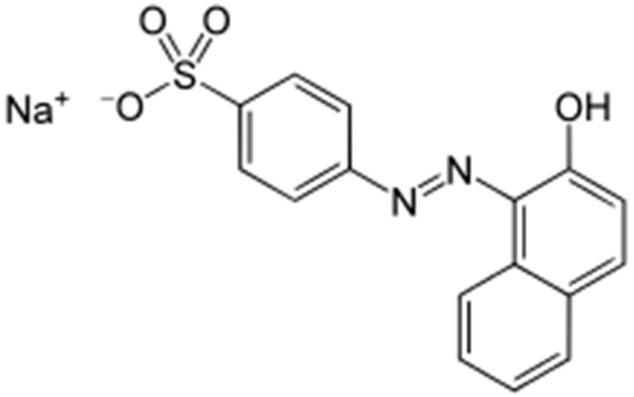

Figure S2. Structure of Acid Orange 7

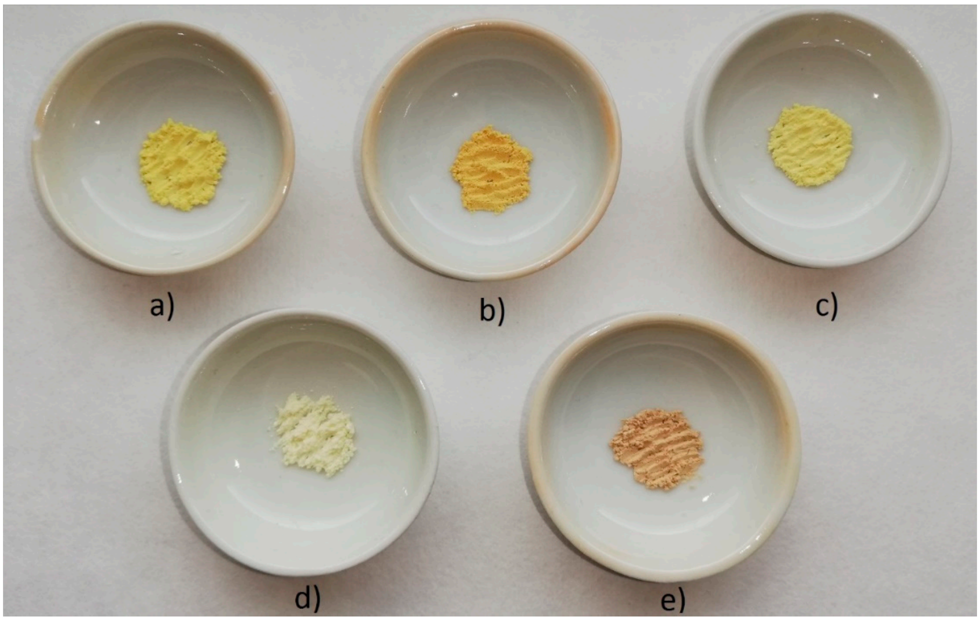

Figure S3. Images of prepared nanomaterials. a) CN, b) S-CN, c) Mes-CN, d) ExCN, e)Mes-ExCN.
